# Effect of auto-adaptive metal artifact reduction (aMAR) program in cone-beam computed tomography on assessing pre-implant bone levels

**DOI:** 10.34172/japid.2024.011

**Published:** 2024-05-28

**Authors:** Farida Abesi

**Affiliations:** Dental Materials Research Center, Health Research Institute, Babol University of Medical Sciences, Babol, Iran

**Keywords:** CBCT, Implant, Metal artifact reduction

## Abstract

This research aimed to introduce an auto-adaptive metal artifact reduction (aMAR) algorithm in cone-beam computed tomography (CBCT) to assess the levels of the pre-implant alveolar crest. Dental implants as a treatment modality for edentulous patients consist of a titanium alloy, which creates a metal artifact, resulting in a dark dental structure in the CBCT scans. Metallic artifacts are limiting factors for the precise detection in CBCT images. These are related to the dark areas around materials and metallic structures (e.g., restorations, implants, and endodontic instruments). To overcome this problem, the metal artifact reduction (MAR) program has been recommended as a post-procedure stage for CBCT image reconstruction. Recent developments offer CBCT scanners with an aMAR option with a greater dynamic range to help overcome the challenges of peri-implant bone evaluation to reach accurate dental diagnoses.

## Dear Editor;

 Dental implants, as a treatment option for edentulous patients, are made of a titanium alloy that creates a metal artifact and makes the dental structures appear significantly darker in CBCT images. CBCT is one of the essential imaging techniques in implant treatment planning and placement.^1^ Cross-sectional images by CBCT provide many advantages, from evaluating the implant site for alveolar crest levels and proximity to critical anatomic structures, including the inferior alveolar nerve and maxillary sinus floor. Therefore, based on these data, the clinicians can plan the surgical procedure accurately to place the implant in the appropriate location.^2^

 Beam-hardening and streak artifacts are limiting factors for the precise detection in CBCT images. These are related to the dark areas around materials and metallic structures (e.g., restorations, implants, and endodontic instruments).^3,4^ The metallic artifact can arise from a combination of many phenomena, such as limitations in the physical process and photon starvation. Metallic objects absorb photons, impeding their detection by the sensor, thereby resulting in artifacts that can compromise the clinical interpretation of CBCT images and data close to the metallic structure. This can lead to poor-quality or non-diagnostic scans and reduced reliability in interobserver and intraobserver assessments.^5^ A notable challenge posed by the lack of data close to the implant fixture is the inability of clinicians to precisely evaluate pre-implant bone levels and the overall health near the bone using CBCT scans.^6^

 To address this problem, the metal artifact reduction (MAR) program has been implemented as a post-procedure stage for CBCT image reconstruction. The MAR tool aims to mitigate artifacts and improve the quality of CBCT images. This algorithm is integrated into the tomographic image reconstruction system to reduce or remove image artifacts. Several companies offer commercial access to these tools for various CBCT equipment, including Newtom (Verona, Italy), Planmeca (Helsinki, Finland), Soredex (Tuusula, Finland), and Vatech (Hwaseong, Korea).^7^

 Various approaches for MAR on CBCT imaging have been examined in previous research. One study involving patients with metallic devices in their bodies demonstrated that applying a pre-processing MAR program can produce superior image quality.^8^ Another study increased the mAs factor or peak tube potential levels, resulting in improved image quality as higher beam energy was not fully absorbed by metallic structures.^9^ Additionally, numerous post-processing techniques for MAR have been investigated, including the multiplanar reconstruction algorithm, which reduced artifacts and enhanced the diagnostic quality of scans.^10^ In the mentioned studies, the rate of metal artifacts was evaluated through visual assessment.

 However, several studies have indicated that the efficiency of the MAR tool may be limited when addressing periodontal and peri-implant defects. Additionally, in some cases, the MAR tool may even lead to a decrease in diagnostic accuracy for detecting root fractures.^7,11^ One limitation of the MAR tool is that it equally removes both the metal artifacts and dental anatomical structures in CBCT images. In recent years, some advancements have been made in post-processing programs to reduce metallic artifacts. For example, recent developments offer CBCT scanners with an auto-adaptive MAR (aMAR) option, which has a greater dynamic range and superior technical specifications that can help overcome the challenges of peri-implant bone evaluation to have accurate dental diagnostics.However, research on applying the aMAR algorithm in CBCT scans remains limited, particularly in cases involving dental implants.^12^

 Moreover, many other approaches have been tested to minimize metal artifacts in CBCT scans and increase detection accuracy in different dental problems.^3^ In this regard, some researchers have proposed that reducing metal artifacts within the field of view, which can increase the overall quality of the images while providing higher spatial resolution,^13^ as well as the CBCT unit and imaging parameters, could influence the formation of artifacts.^14^ On the other hand, several studies have examined the metallic artifacts generated by different dental implant materials, such as ceramic, titanium, and zirconia, in CBCT images.^1,15,16^ For instance, research by Demirturk Kocasarac et al^15^ indicated that implants containing titanium, whether pure titanium or a titanium-zirconia combination, exhibited fewer artifacts compared to zirconia implants. Similarly, Sancho-Puchades et al^17^ found that ZrO_2_ implants resulted in a threefold increase in metallic artifacts compared to titanium implants. However, despite these efforts, none of these methods could eliminate the noise induced by metallic objects in CBCT scans. Therefore, it is imperative to explore factors that could contribute to improved image quality by minimizing image artifacts.

 In contemporary radiology, there is a growing emphasis on obtaining accurate data quickly and automatically. The accessibility of source codes and datasets in deep learning has attracted significant attention. Consequently, numerous studies have evaluated the diagnostic efficacy of integrating artificial intelligence with CBCT imaging for identifying oral and maxillofacial anatomical landmarks and lesions. Researchers have reported differing degrees of accuracy in these assessments.^14^ On the other hand, there is limited research on evaluating different clinical reconstruction algorithms and networks in the aMAR program and CBCT imaging technique. Moreover, there are some gaps in our knowledge about assessing the clinical applications and diagnostic performance of aMAR in dental and maxillofacial radiology, describing the value, impact, and reliability of aMAR in CBCT scans, especially in the case of multiple implants, which would benefit from further research.

## Competing Interests

 None declared.

## Ethical Approval

 Not applicable.

## Funding

 Not applicable.
